# Response of Electrical Activity in an Improved Neuron Model under Electromagnetic Radiation and Noise

**DOI:** 10.3389/fncom.2017.00107

**Published:** 2017-11-21

**Authors:** Feibiao Zhan, Shenquan Liu

**Affiliations:** School of Mathematics, South China University of Technology, Guangzhou, China

**Keywords:** Morris-Lecar model, electromagnetic induction, Gaussian white noise, electrical activity, bursting, bifurcation

## Abstract

Electrical activities are ubiquitous neuronal bioelectric phenomena, which have many different modes to encode the expression of biological information, and constitute the whole process of signal propagation between neurons. Therefore, we focus on the electrical activities of neurons, which is also causing widespread concern among neuroscientists. In this paper, we mainly investigate the electrical activities of the Morris-Lecar (M-L) model with electromagnetic radiation or Gaussian white noise, which can restore the authenticity of neurons in realistic neural network. First, we explore dynamical response of the whole system with electromagnetic induction (EMI) and Gaussian white noise. We find that there are slight differences in the discharge behaviors via comparing the response of original system with that of improved system, and electromagnetic induction can transform bursting or spiking state to quiescent state and vice versa. Furthermore, we research bursting transition mode and the corresponding periodic solution mechanism for the isolated neuron model with electromagnetic induction by using one-parameter and bi-parameters bifurcation analysis. Finally, we analyze the effects of Gaussian white noise on the original system and coupled system, which is conducive to understand the actual discharge properties of realistic neurons.

## 1. Introduction

Neural network is composed of a large number of neurons and the connection between neural networks is through signal propagation between neurons such as chemical or electrical signal. Neurodynamics researchers really pay much attention to dynamical properties of electrical activity in neurons or neural networks starting from the establishment of a reliable Hodgkin-Huxley (Hodgkin and Huxley, 1952) model that is established by describing ion channels in neurons. Based on the Hodgkin-Huxley model, it is extensively explored that many neurons have own model of ion channels and even a neuron may have multiple models. For example, the dimensionless Hindmarsh-Rose (Hindmarsh and Rose, 1984) model is a mathematical model and it has many firing behaviors of neurons by adjusting system parameters or external forcing stimuli such as bursting, spiking, quiescent and chaotic states. It can examine transition mode of electrical activity by using bifurcation analysis (Storace et al., 2008) and its mathematical properties are analyzed in Liu and Liu (2012) in detail. Although minimized Morris-Lecar model (Morris and Lecar, 1981) is consists of two coupled first-order differential equations, it still has a variety of discharge activities and describes the nature of the barnacle giant muscle fiber. It is further investigated by improving the neuron model (Duan et al., 2010; Wang et al., 2011; Upadhyay et al., 2017) or using theoretical analysis (Tsumoto et al., 2006). In addition to these, a variety of simplified neuron model (Izhikevich, 2004; Ibarz et al., 2011) have been developed and used for theoretical and numerical exploration.

With the development of neural dynamics, investigators propose some ways to deeply research mathematical mechanism of neuron model. For example, Izhikevich (2000) make a classification of electrical activity of low-dimensional neurons using slow-fast dynamics analysis (Rinzel, 1987; Rinzel and Ermentrout, 1998). And Szmolyan and Wechselberger (2001) argue that canard theory is used to illustrate mixed-mode oscillations mechanism in neurons. Based on these ways, some explorations are more detailed via combining them with other valid research methods and there is a widely extension in specific neuron model (Gu et al., 2003, 2012; Wang et al., 2015, 2017; Lu et al., 2016; Li and Gu, 2017; Zhao and Gu, 2017). Gu and Pan (2015) determine improved neuron model and use different ion currents to discuss its bifurcation behaviors. Furthermore, some experimental works is presented in Gu et al. (2013a, 2014) and they further investigate transition mode of electrical activity using bifurcation analysis. Some authors also examine the effects of noise on the coherence resonance, stochastic resonance and firing behavior of the neuron model (Gu et al., 2013b; Jia and Gu, 2015a,b; Wang et al., 2016; Wang and Ma, 2017) in addition to adding external forcing stimulus to the neuronal system. In fact, time-varying noise increases the system's dimensions, which is also a way to explore dynamical behaviors of neuron model and it can restore authenticity of neuronal system. Recently, energy-coded neurons are proposed to understand firing behavior of neurons as a new perspective and it is discussed in Wang et al. (2009), which define a Hamilton energy and Song et al. (2015) suggest that the Hamilton energy may be higher when neuron is in the spiking states rather than bursting or chaos states. Some researchers Li et al. (2016) investigate the discharge behaviors by adding equivalent current to electromagnetic radiation in neuronal loop. Yi et al. (2012, 2015) show that spiking pattern and spiking-frequency of neurons are changed when neurons are exposed to an electric field. Therefore, the effect of electromagnetic induction on neurons or neural networks may need to be considered to set more authentic neuron model.

As reported in Lv and Ma (2016) and Lv et al. (2016), we will also use magnetic flux to describe the effect of electromagnetic induction and we further explore electrical activity of neuronal system when it is exposed to electromagnetic radiation. However, they present these results without considering noise system such as Levy noise or phase noise. In this paper, the effect of electromagnetic radiation is explored on the Morris-Lecar neuron model and phase noise is also implemented to investigate resonance mode. Specifically, we change one of them to examine dynamical behavior of electrical activity when electromagnetic radiation and noise are added to the original system at the same time. In addition, we examine bursting transition mode via presenting one-parameter bifurcation diagram and bi-parameter bifurcation diagram when only adding electromagnetic induction to the original neuronal system. Finally, we compare the electrical behavior of isolated neurons with that of coupled neurons when only adding Gaussian white noise to respective system.

## 2. Model and methods

We use an improved M-L neuron model, which is reported in the previous investigation and it is a real biological neuron model which describes the giant barnacle muscle fiber. As we known, its dynamical behavior is greatly abundant although the model contains only calcium ion channel and potassium ion channel. Two variables are membrane voltage *V* and activated gating channel *n* in the original model. The improved neuron model contains five first-order differential equations, which is described as follows:

(1) $$\begin{array}{l} \left\{ \begin{array}{l} C\frac{dV}{dt}=-I_{Ca}-I_{K}-I_{L}-k\rho \left(\varphi \right)V+I_{D}+I,\\ \frac{dn}{dt}=\phi \frac{n_{\infty }-n}{\tau_{n}},\\ \frac{dI}{dt}=\varepsilon \left(V_{0}-V\right),\\ \frac{d\varphi }{dt}=k_{1}\nu -k_{2}\varphi ,\\ \frac{dQ}{dt}=\omega_{1}+\sqrt{2D}\xi \left(t\right). \end{array}\right. \end{array}$$

(2) $$\begin{array}{l} \left\{ \begin{array}{l} I_{Ca}=g_{Ca}m_{\infty }\left(V\right)\left(V-V_{Ca}\right),m_{\infty }=\frac{1}{2}\left(1+\tanh\left(\frac{V-V_{1}}{V_{2}}\right)\right),\\ I_{K}=g_{K}n\left(V\right)\left(V-V_{K}\right),n_{\infty }=\frac{1}{2}\left(1+\tanh\left(\frac{V-V_{3}}{V_{4}}\right)\right),\\ I_{L}=g_{L}\left(V-V_{L}\right),I_{D}=A_{1}\sin\left(Q\left(t\right)\right),\\ \rho \left(\varphi \right)=\alpha +3\beta \varphi^{2},\tau_{n}=1/\cosh\left(\frac{V-V_{3}}{2V_{4}}\right). \end{array}\right. \end{array}$$

Where *I*_*Ca*_, *I*_*K*_, *I*_*L*_, *I*_*D*_, and *I* are inward calcium current, potassium current, leakage current, phase noise, and voltage-dependent feedback current, respectively. The variables *V*, *n*, φ represent membrane potential, gate variable for potassium channel and magnetic flux across membrane during the process of ion transfer, respectively. *I* is a feedback current, which is very sensitive to membrane potential. The function ρ(φ) is associated with magnetic flux, readers can refer to Bao et al. (2010), Wu et al. (2016), Wu F. et al. (2017); Wu J. et al. (2017), and Xu et al. (2017) to obtain more detailed information. ξ(*t*) is Gaussian white noise, ω_1_, *A*_1_ are angular frequency and amplitude for forcing currents, *Q*(*t*) is phase noise. Detailed system parameters are explained as: membrane capacitance *C*, maximal conductance *g*_*Ca*_, *g*_*K*_, *g*_*L*_, reversal potential *V*_*Ca*_, *V*_*K*_, *V*_*L*_, the other kinetics parameter ϕ, ε, *V*_1_, *V*_2_, *V*_3_, *V*_4_. Some parameters of electromagnetic radiation are α, β, *k*, *k*_1_, *k*_2_. Readers can refer to Morris and Lecar (1981) and Ma et al. (2017) to understand the meaning of these symbols and there are detailed data in Lv et al. (2016). Specific parameters throughout the paper are given in Table A1. Furthermore, coefficient variability (abbreviated as CV and labeled as η) of interspike intervals (ISIs) sequence (Pikovsky and Kurths, 1997) is presented as the radio of standard deviation(*std*) of ISIs sequence to its mean, i.e., η = *std*(*ISIs*)/*mean*(*ISIs*), and it indicates the coherence degree. In section 4, we are using electrical coupling between neurons, i.e., the coupling term is as follows: *I*_*couple*_ = *g*_*c*_(*V*_1_ − *V*_2_), where *g*_*c*_ represents the coupling coefficient and *V*_1(2)_ denote the membrane potential of one(another) of neuron.

In fact, we are familiar with the M-L model, but we still have a novel understanding and discovery for the improved M-L model via adjusting electromagnetic induction and phase noise. In this paper, we adopt fourth-order Runge-Kutta algorithm to exhibit numerical solution of the neuronal system with time step *d* = 0.01 *ms* in all of simulation and calculation, and we use MATPLOTLIB software package in PYTHON for all numerical calculation and graphic rendering.

## 3. Multiple discharge behaviors under electromagnetic radiation

In this section, we discuss that amplitude *A*_1_ is how to adjust electricity activity via changing the range of forcing amplitude, and we also examine firing pattern of improved M-L neuron model by adjusting magnetic flux parameter *k*. Moreover, we also calculate coefficient variability of interspike intervals sequence to explore the coherence degree by altering noise intensity *D* and it represents that the smaller the CV value is associated with a better coherence. Furthermore, we only consider the system with electromagnetic induction and without Gaussian white noise. In this process, we find that the effect of electromagnetic field on the neuronal system is two-sided. The detailed analysis is as follows.

**Figure 1:**
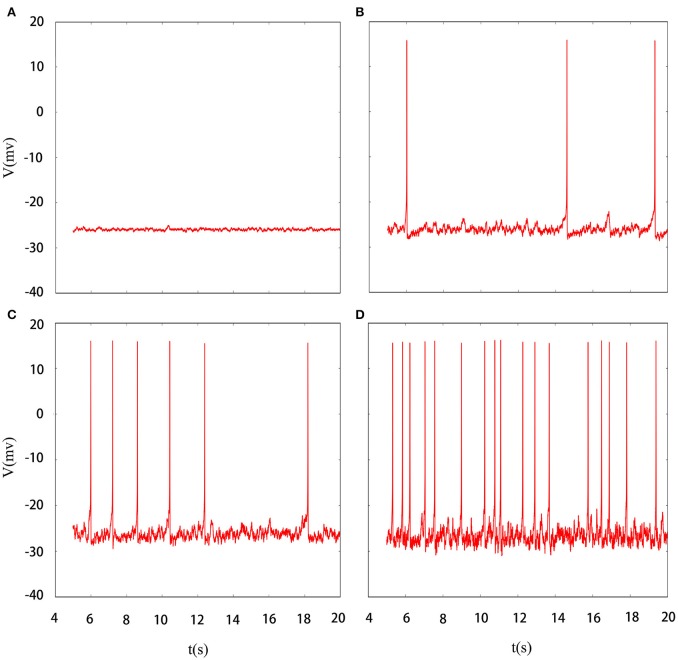
Time series of membrane potential under different amplitudes. **(A)** *A*_1_ = 1, **(B)** *A*_1_ = 3, **(C)** *A*_1_ = 5, **(D)** *A*_1_ = 10, the noise intensity is selected as *D* = 10, ϕ = 0.22, *k* = 0.0025, ω_1_ = 0.5.

As shown in Figure 1, electrical activity of improved neuron model can be controlled and adjusted as a random but relatively stable pattern. Specifically, the system is in a relative resting state when *A*_1_ = 1 and the firing occurs when we increase *A*_1_ to 3. We find that the number of spiking is gradually increased when we continue to increase amplitude from 3 to 5. It is described that the amplitude of phase noise makes a positive response to the system although the range of amplitude is small. Certainly, this process is reversible. That is to say, we can better adjust the spiking of neurons by regulating the amplitude so that neuronal model is more reasonable in the simulation of realistic neural network.

**Figure 2:**
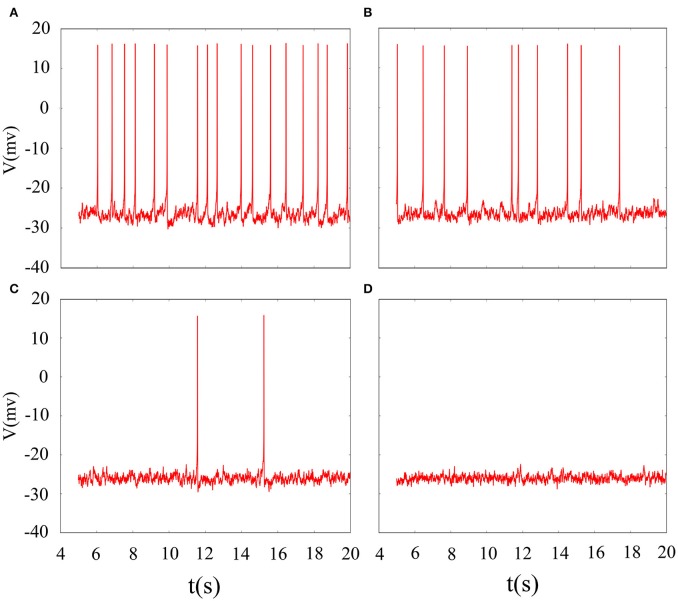
Time series of membrane potential under different feedback coefficient. **(A)** *k* = 0.002, **(B)** *k* = 0.0025, **(C)** *k* = 0.003, **(D)** *k* = 0.0035, the noise intensity is controlled as *D* = 10, ϕ = 0.22, *A*_1_ = 6, ω_1_ = 0.5.

**Figure 3:**
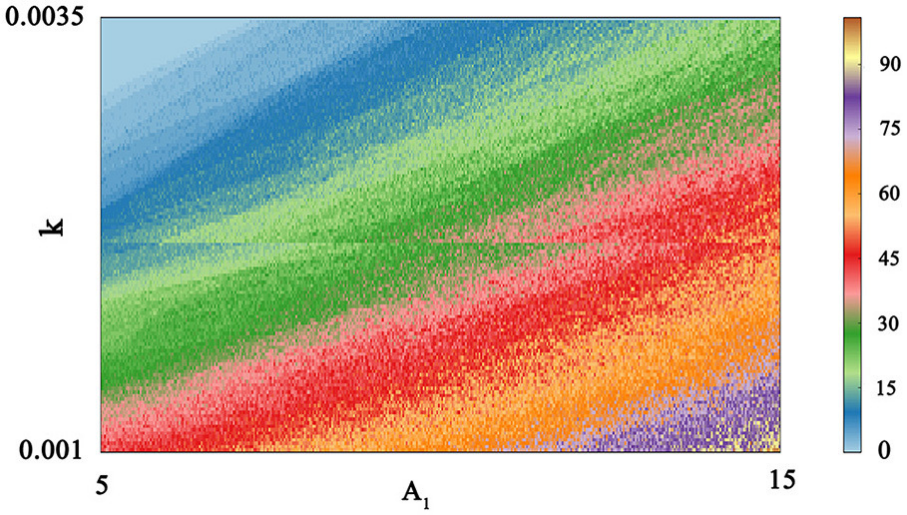
Bi-parameter bifurcation diagram of suprathreshold spiking. The number of suprathreshold spiking is presented at the right sides by the colorful belt and 0 indicates the relatively resting state.

Indeed, multiple patterns of electrical activity are detected in the improved neuron model by modifying the feedback coefficient *k*, which indicates the coupling strength between magnetic flux and membrane potential. It is found that magnetic flux has a great effect on the membrane potential so that multiple modes can be selected with the changing of feedback coefficient, and time sequence of membrane potential is shown in Figure 2. We also explain the discharge process in detail. First, suprathreshold spiking of system is gradually reduced. when feedback coefficient *k* = 0.002, the number of suprathreshold spiking is more than *k* = 0.0025. It is in a relatively resting state (subthreshold spiking) when *k* increase to 0.003. From above analysis, we can see that firing activity of membrane potential is more dependent on the feedback coefficient, which is greatly sensitive in regulating membrane potential. Next, we will consider their combined effect on neuronal suprathreshold spiking by calculating bi-parameter bifurcation diagram, which is shown in Figure 3. It is exhibited on the (*k, A*_1_) plane and presents a detailed suprathreshold change.

**Figure 4:**
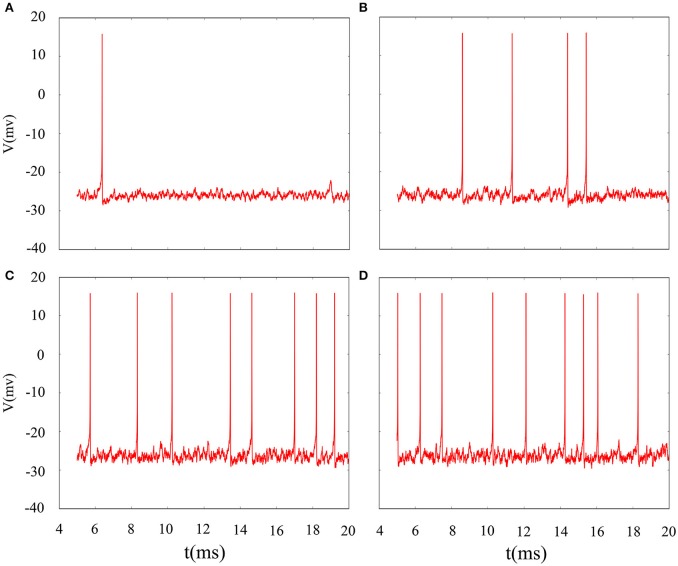
Time series of membrane potential under different noise intensity *D*. **(A)** *D* = 1, **(B)** *D* = 3, **(C)** *D* = 5, **(D)** *D* = 10, the feedback coefficient is controlled as *k* = 0.0025, *A*_1_ = 6, ϕ = 0.22, ω_1_ = 0.5.

**Figure 5:**
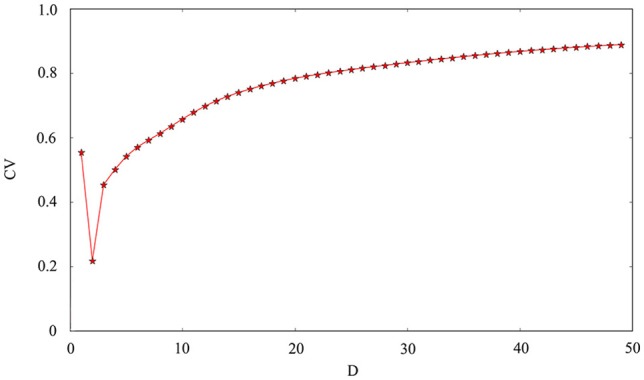
Coefficient variability of ISIs series of membrane potential. The abscissa is noise intensity *D* and the ordinate is the *CV*. Other controllable parameters are given as *k* = 0.0025, *A*_1_ = 6, ϕ = 0.22, ω_1_ = 0.5.

The phase noise is induced by differential equations with Gaussian white noise and it can be grasped by noise intensity *D*. Gaussian white noise has a good simulation of noise system in the real neural network so that discharge activity is more authentic. Therefore, it is necessary that we will incorporate noise into neuron model to simulate computation. Figure 4 exhibit time sequence of membrane potential of dynamical system, which is an improved neuron model with different noise intensity *D*. Especially, it is interesting that multiple oscillation modes are strengthened with the increasing of noise intensity. From the diagram, we can see that noise promote the spiking of neuronal system to some extent but it is two-sided. Its effect may be more regular on the spiking than changing amplitude or adjusting feedback coefficient. For the neuronal system, coefficient variability of ISIs sequence is often used to examine its coherence degree. Readers can refer to Jia and Gu (2015a) and Pikovsky and Kurths (1997) to get a more detailed understanding about the concept of CV. It indicates that the better coherence depends on the smaller the CV. As shown in Figure 5, it will has a good coherence in a suitable noise intensity *D*, and that is to say, the distance is uniform between spiking and spiking at this moment. The CV value starts to increase when noise is greater than that fixed value *D* = 2. In other words, appropriate noise may produce a positive response to the neuronal system.

**Figure 6:**
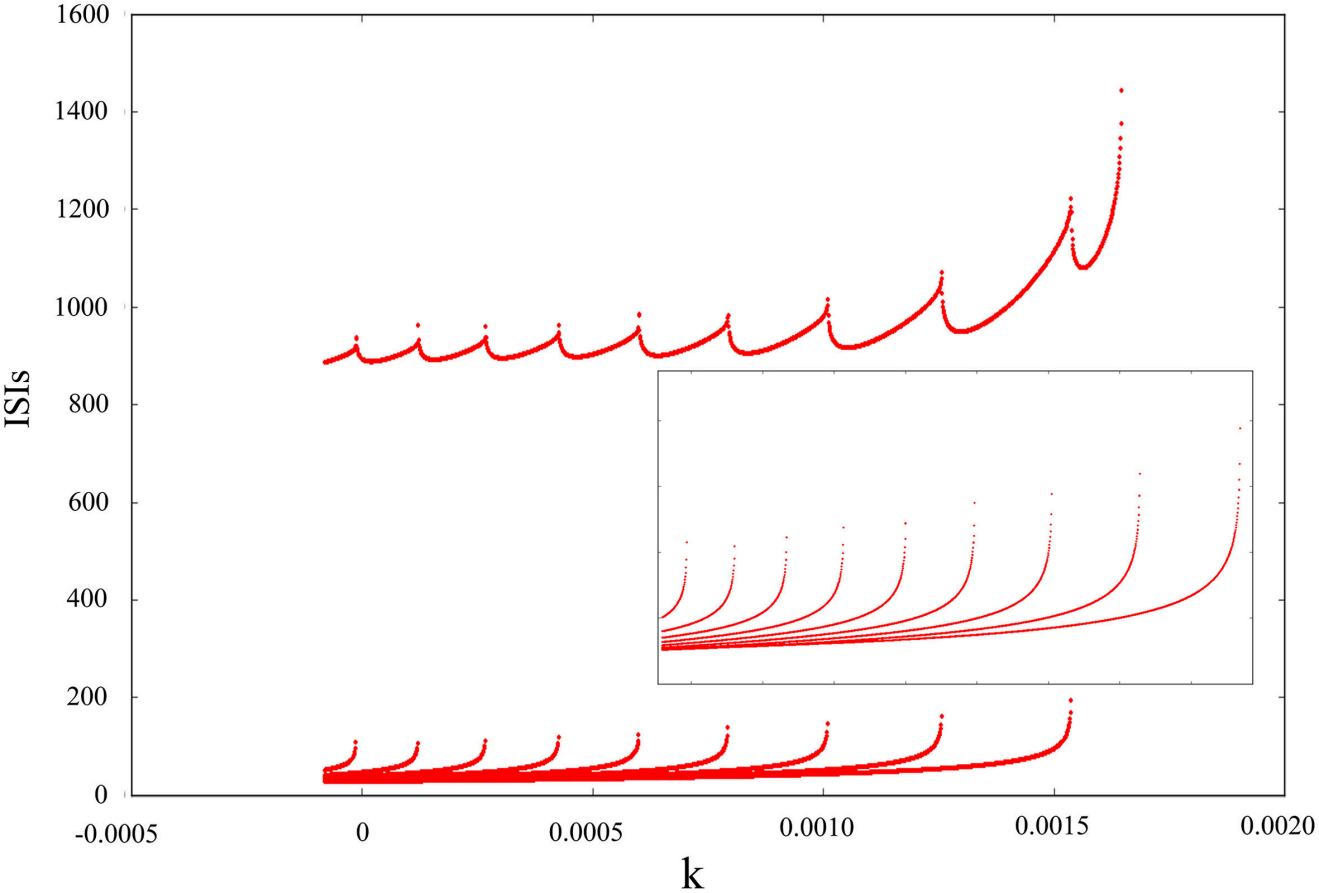
Interspike intervals for time series of membrane potential with the increasing of feedback coefficient *k*, and no Gaussian white noise is added.

The above discussion is based on adding noise and electromagnetic field at the same time and then changing amplitude, feedback coefficient or noise intensity to obtain some firing behaviors of dynamical system. Next, we will discern the oscillating mode only adding electromagnetic radiation or adding Gaussian white noise. The result in Figure 6 convey that the oscillating behavior can be regulated and adjusted as an isolated pattern. In other words, appropriate mode can be selected by adding suitable magnetic flux on membrane potential. From the diagram, we can see that inverse period-adding bifurcation is observed with the increasing of feedback coefficient *k* and this bifurcation diagram is regular, but it eventually jumps to resting state. Obviously, we get a lot of oscillation modes of discharge activity via changing one-parameter, and it makes us very interested in the adjustment of bi-parameter.

**Figure 7:**
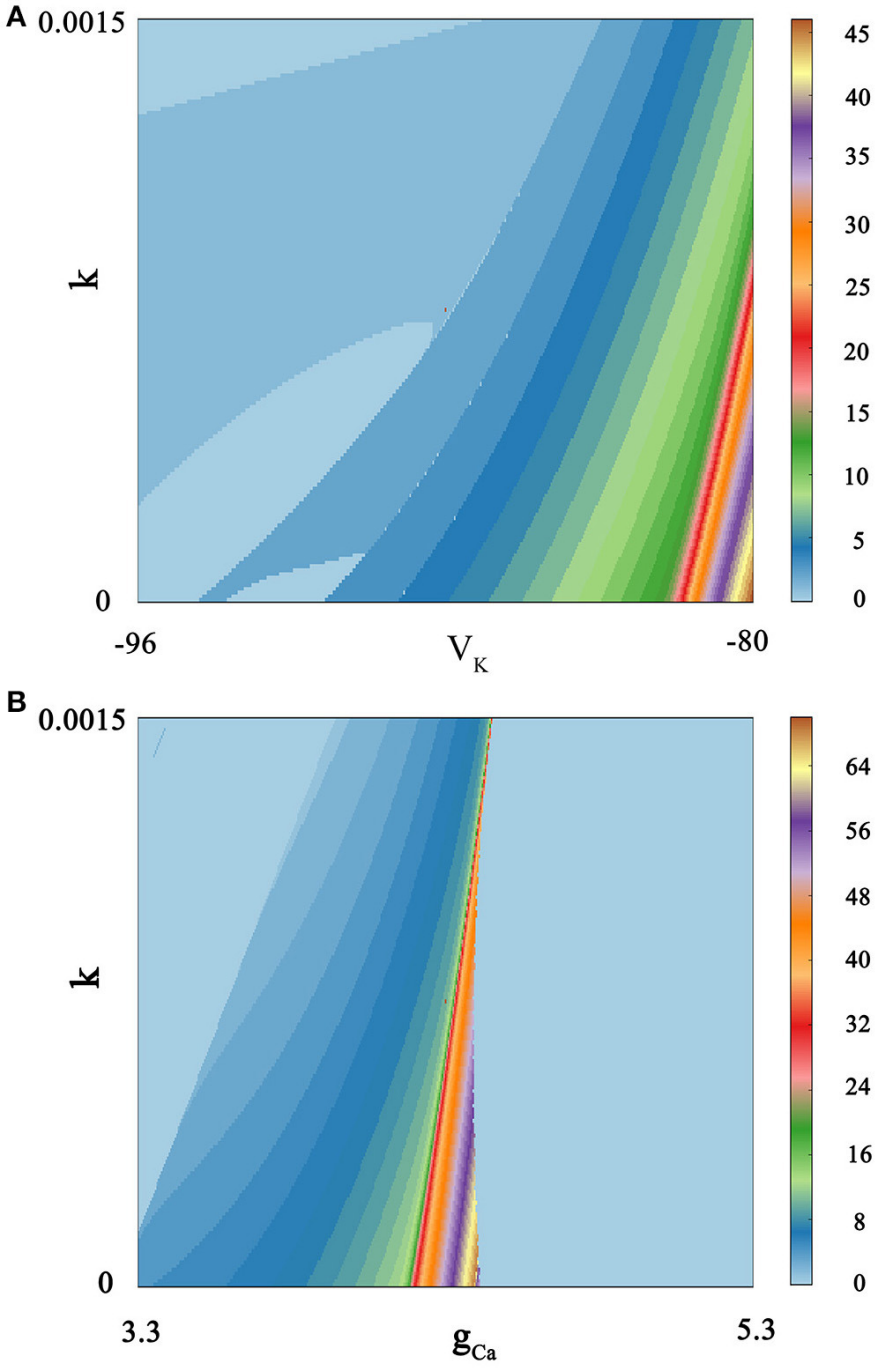
Spiking-counting diagram as the change of bi-parameter. The number of spiking per bursting is exhibited at the right sides by the colorful belt. And no Gaussian white noise is considered. **(A)** (*k, V*_*K*_) plane; the number 0 indicates quiescent condition and the number 1 represents tonic spiking whilst the number 2–45 denotes regular bursting; **(B)** (*k, g*_*Ca*_) plane; similarly, the number 0 indicates quiescent condition and the number 1 represents tonic spiking; the number 2–69 denotes regular bursting whilst the number 70 indicates chaotic states.

We calculate bi-parameter bifurcation diagram and show it in Figure 7. In Figure 7A, where the abscissa represents the reversal potential *V*_*K*_ whose value is taken from −96 to −80, and the ordinate denotes the feedback coefficient *k* whose value is selected from 0 to 0.0015. Moreover, the color scale bar indicates a gradual process, which represents the number of spiking per burst from the regular bursting to the chaotic bursting. Examining the Figure 7A, we can see that the number of spiking per burst will continue to increase with the changing of bi-parameter until it reaches chaos in a small range of lower right corner. In addition, the spiking phenomenon can occur between two resting state. As shown in Figure 7B, we exhibit bi-parameter bifurcation diagram in the (*k, g*_*Ca*_) plane, where the abscissa indicates the maximal conductance *g*_*Ca*_ whose value is taken from 3.3 to 5.3, and the ordinate represents the feedback coefficient *k* whose value is selected from 0 to 0.0015. We can see that triangular area on the left side of the diagram and trapezoidal area on the right are two resting states. And it is obvious that the spiking go through the transition process from regular bursting to chaotic bursting and then to regular bursting by adjusting these two parameters. The electrical activity is very sensitive to bi-parameter because most bursting modes have only narrow strip areas.

By comparing Figure 7A with Figure 7B, it is easy to see that these two parameters have a great influence on the discharge activity. For the same feedback coefficient interval, if we change the reversal potential *V*_*K*_ at the same time, we will get a continuous spike-adding mode. But if we transform the maximal conductance *g*_*Ca*_, we will see that electrical activity will return to period bursting via chaotic bursting. The principle is hidden behind a large number of patterns, and it can be well discerned by simulating the neuronal system.

## 4. Sensitivity of coupled neurons

In this section, we will discuss dynamical response of an isolated neuron and two coupled neurons. These two systems are exposed to Gaussian white noise, and they have many different responses to the noise system. In addition, we examine how the noise affects coupled neuronal system and compare the discharge activity of coupling neuronal system with that of original coupling system without noise. Furthermore, we may find some new phenomena by comparing electrical mode of isolated neurons with that of coupled neurons under noise, and we also present a new perspective to explore neurons responding to noise. They are analyzed as follows.

**Figure 8:**
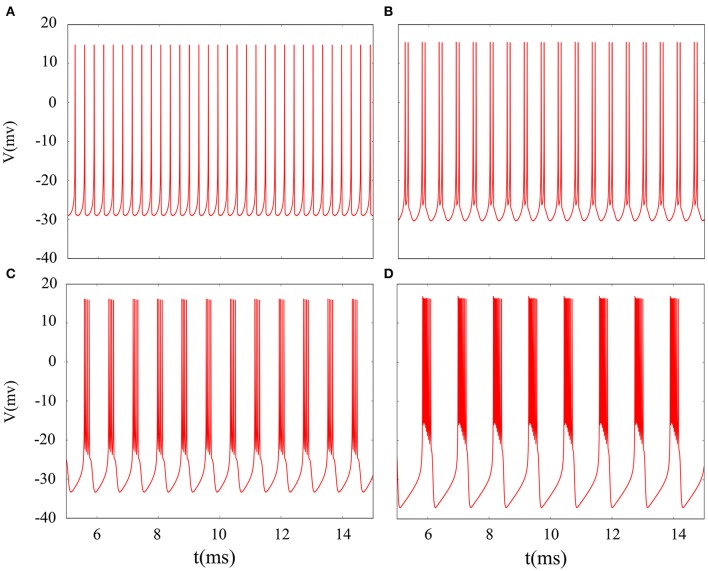
Time series of membrane potential under different reversal potential *V*_*K*_. **(A)** *V*_*K*_ = −96, **(B)** *V*_*K*_ = −92, **(C)** *V*_*K*_ = −88, **(D)** *V*_*K*_ = −84, and no noise is introduced.

**Figure 9:**
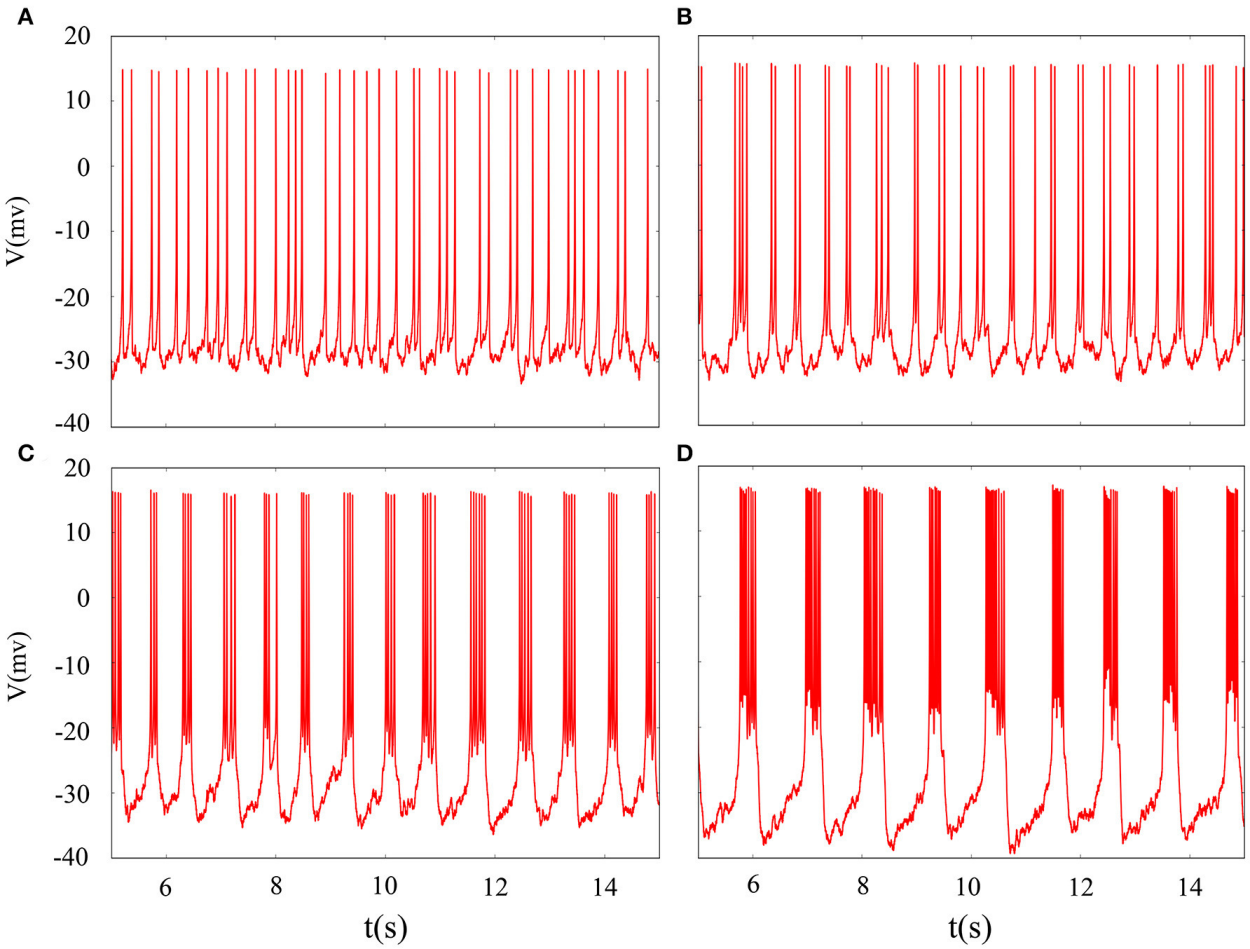
Time sequences of membrane potential under different reversal potential *V*_*K*_. **(A)** *V*_*K*_ = −96, **(B)** *V*_*K*_ = −92, **(C)** *V*_*K*_ = −88, **(D)** *V*_*K*_ = −84, and Gaussian white noise is considered.

It is inevitable that many realistic neuron system will be exposed to noisy environment and we find that the effect has two-sided. Therefore, we will explore neuronal system by adding the noise to neuron model without adding electromagnetic radiation. Figure 8 indicates time series of membrane potential without adding noise, and it is tonic spiking or regular bursting. Figure 9 represents time series of membrane potential after adding noise system and corresponding to Figure 8, respectively. Observing the diagram, we can discern that noise is very sensitive to the tonic spiking and its response is more intense than the regular bursting. In detail, electrical activity of tonic spiking is greatly disturbed and period-2 bursting is also disrupted. In addition, regular period-4 bursting and square wave busting have also slight perturbation and become irregular discharge mode. In general, the more the number of spiking per burst means that the mode is relatively more stable when adding noise to system. Moreover, bursting may be increased during the same time period when Gaussian white noise is introduced into the neuron system whilst the number of spiking per burst will also be fluctuant and most of them is increased. Furthermore, we can see that noise has a little control over the firing state of neuron but it may has a great effect on bistable state (down resting state and upper steady state). As we known, bursting may mean more neural information than spiking. To sum up, we may be able to infer that the more complex discharge activity is better anti-interference than tonic spiking, and this may be associated with the robustness of neural networks.

**Figure 10:**
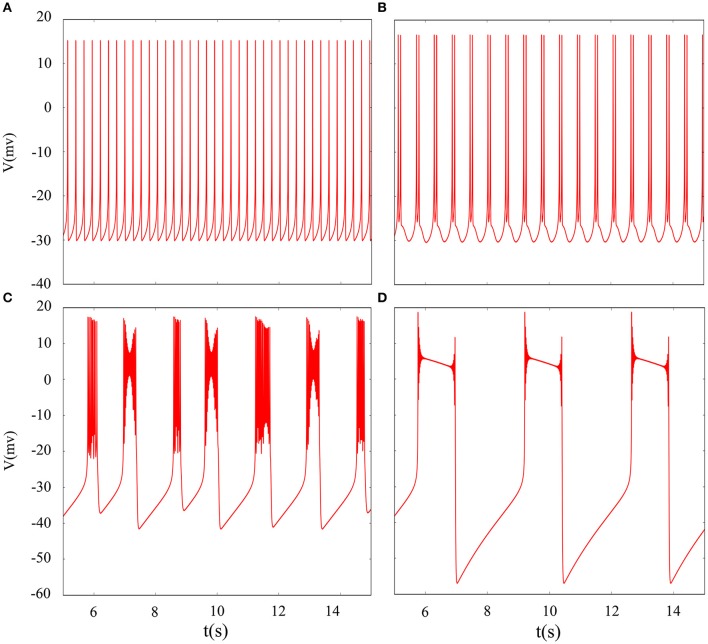
Time series of membrane potential of coupled neurons under different reversal potential *V*_*K*_. **(A)** *V*_*K*_ = −92, **(B)** *V*_*K*_ = −88, **(C)** *V*_*K*_ = −84, **(D)** *V*_*K*_ = −76, and adding Gaussian white noise.

**Figure 11:**
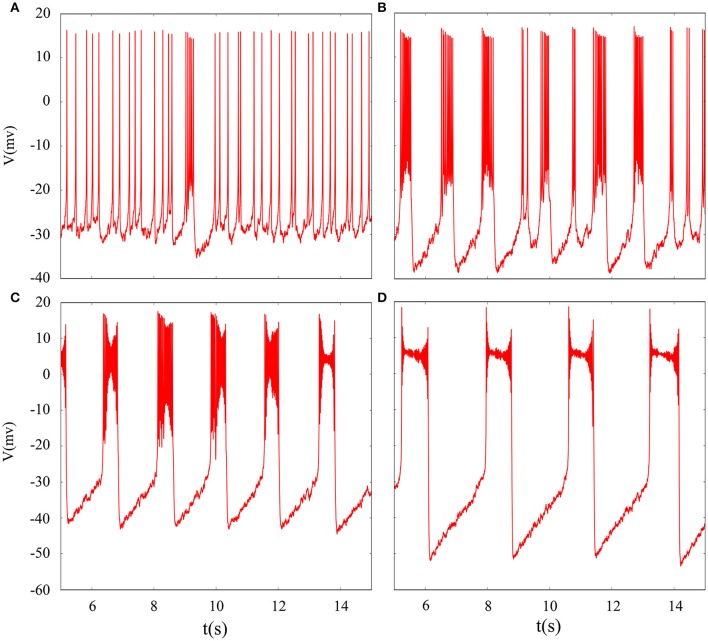
Time series of membrane potential of coupled neurons under different reversal potential *V*_*K*_. **(A)** *V*_*K*_ = −92, **(B)** *V*_*K*_ = −88, **(C)** *V*_*K*_ = −84, **(D)** *V*_*K*_ = −76, and Gaussian white noise is considered.

In fact, we have explored multiple modes of discharge activity in coupled neuron system by changing reversal potential and adding Gaussian white noise to system and it has more patterns than in an isolated neuron. Therefore, we are interested in the coupling system with noise. Figure 10 represents time series of membrane potential without adding noise, which are tonic spiking, regular bursting and irregular bursting. Figure 11 exhibits time series of membrane potential under noise and corresponding to Figure 10, respectively. Comparing two diagram, we find that the effect of noise on the spiking is greater than bursting and this is consistent with our analysis of single neurons. In noise system, there is a bursting that appears in the tonic spiking and period-2 bursting become many irregular bursting (the number of spiking per burst is not less than two). Moreover, it is also disrupted that bursting alternately appears in Figure 10C and it becomes unstable bursting mode. Furthermore, the change once again confirms that the effect of noise on bistable state is stronger than firing state as shown in Figure 10D. In addition, the effect of noise on down resting state of coupled system is obviously greater than that of upper steady state, but this feature is not obvious in an isolated neuron model. These are new phenomena and not present in a single neuron. It is not the same as in an isolated neuron that the noise does not increase the number of bursting during the same time (Contrast Figure 10C and Figure 11C), but the main effect of noise on the bistable state is retained. Therefore, we may be able to suggest that the exploration of neural network under noise requires a long-term process to achieve an in-depth level and this process is valuable.

## 5. Conclusions

Tonic spiking and bursting are effective coding of signals between neurons and abundant discharge activity patterns are accompanied by complex signal propagation. The effects of electromagnetic radiation and noise on discharge activity of system are two-sided and it has been investigated by some researchers and their co-workers (Lv et al., 2016; Ma et al., 2017). Therefore, we are more interested in electrical activity of system with electromagnetic induction or noise and hope that it is controllable. In this paper, we have explored transition of electrical activity in an improved Morris-Lecar neuron model, which contains electromagnetic radiation and noise. Additionally, electromagnetic radiation is expressed through the magnetic flux and noise obeys normal distribution. By comparing the simulation of the system, we have found that there is a great change in discharge activity until external forcing stimulus is removed from system and dynamical response will be more sensitive by adjusting more bifurcation parameters. One of these, we have calculated transition of discharge activity with the changing of the feedback coefficient *k*. In addition, we have examined the effect of noise intensity D on coherence of electrical activity via describing the changes of coefficient variability. Furthermore, we have drew interspike intervals sequence diagram and bi-parameters bifurcation diagram to argue the effect of electromagnetic induction on the fluctuation of membrane potential. Finally, we have compared the response of noise on the electrical activity of isolated neurons with that of coupled neurons. From the above, we know that electromagnetic induction and noise can arouse different dynamical behaviors of neuronal systems. Logically, we will try to illustrate their impact on the biological meaning of real neurons. In fact, move of charged ions involves a small and easily overlooked magnetic field during the process of ion transmembrane movement. But it may be that accumulation of these small induced currents produces qualitative change in a single neuron or even in a neural network. Certainly, the effect of noise on the neuronal system is also often explored, since many realistic neural network are in a noisy environment. Therefore, we may be able to consider incorporating the magnetic flux into real neuron system because of its impact on discharge activity and noise can not be ignored. According to the simulation of the improved neurons, external bifurcation parameters can change firing mode, which may mean that neurons can select appropriate electrical behaviors due to its self-adaption that frequently appears in neural networks.

## Author contributions

The idea of this article is proposed by SL and the specific writting is done by FZ.

### Conflict of interest statement

The authors declare that the research was conducted in the absence of any commercial or financial relationships that could be construed as a potential conflict of interest.

## Appendix

**Table A1:** Fixed parameter values are used in the calculation.

**Definition**	**Parameter values**
Reversal potentials (mv)	*V*_*Ca*_ = 120, *V*_*K*_ = −84, *V*_*L*_ = −60
Maximal conductance (ms/*cm*^2^)	*g*_*Ca*_ = 1.0, *g*_*K*_ = 8.0, *g*_*L*_ = 2.0
Gating variable parameters (mv)	*V*_1_ = −1.2, *V*_2_ = 18, *V*_3_ = 12, *V*_4_ = 17.4, *V*_0_ = −26
Other dynamical parameters	*C* = 20μ*f*/*cm*^2^, ϕ = 0.23, ε = 0.001
Electromagnetic induction parameters	α = 0.1, β = 0.02, *k* = 0.0025, *k*_1_ = 0.9,
	*k*_2_ = 0.5, ω_1_ = 0.5, *A*_1_ = 6, *D* = 10
